# Insulin resistance and associated factors in patients with Type 1 Diabetes

**DOI:** 10.1186/1758-5996-6-131

**Published:** 2014-12-01

**Authors:** Mônica Maria Teixeira, Maria de Fátima Haueisen Sander Diniz, Janice Sepúlveda Reis, Teresa Cristina Abreu Ferrari, Maria Goretti Bravim de Castro, Bruna Polonio Teixeira, Isabella Cristina da Silva Arantes, Danielle Marques Bicalho, Rodrigo Bastos Fóscolo

**Affiliations:** Federal University of Minas Gerais UFMG, Rua Padre Rolim 769, sala 802. Bairro São Lucas, Belo Horizonte, Minas Gerais Brazil; Department of Internal Medicine, UFMG, Belo Horizonte, Minas Gerais Brazil; Endocrinology Service, Santa Casa of Belo Horizonte, Belo Horizonte, Minas Gerais Brazil; Centre for Studies in Densitometry, Belo Horizonte, Minas Gerais Brazil

**Keywords:** Type 1 diabetes, Insulin resistance, Estimated glucose disposal rate formula, Insulin sensitivity score

## Abstract

**Objective:**

To assess the presence of insulin resistance (IR) in patients with type 1 diabetes (T1DM) according to the estimated glucose disposal rate formula (eGDR) and the insulin sensitivity score (ISS) and to estimate the correlation between these two measures and identify the clinical and laboratory markers related to IR.

**Research design and methods:**

Cross-sectional study of adults with T1DM (n = 135). The results of the formulas that estimate IR were separated into quartiles and correlated with demographic data, clinical characteristics and laboratory parameters. We analyzed the total and regional adiposity by dual-energy X-ray absorptiometry and skin fold thickness measurements.

**Results:**

Two thirds of the patients were overweight or obese. A moderate correlation was found between eGDR and ISS (r = 0.612). The results of both formulas were positively correlated with BMI (r = −0.373 eGDR and r = −0.721 ISS), thoracic-abdominal fat (r = −0.484 eGDR and r = −0.758 ISS), waist/height ratio (r = −0.537 eGDR and r = −0.779 ISS), subscapular skinfold (mm) (r = −0.356 eGDR and r = −0.569 ISS), total dose insulin IU/lean mass (kg) (r = −0.279 eGDR and r = −0.398 ISS), age (years) (r = −0.495 eGDR and r = −0.190 ISS) and diabetes duration (years) (r = −0.428 eGDR and r = −0.187 ISS).

A moderate agreement (Kappa 0.226) was observed between the 1st quartile of results determined by the formulas in 10.4% of the patients, but the 4th quartile presented a strong correlation (Kappa 0.679). The individuals with IR that were classified in the 1st quartile by the ISS formula had a higher chance of presenting with *acanthosis nigricans* (OR = 5.58, 95% CI =1.46-21.3).

**Conclusions:**

The correlations found in this study indicate the possibility of using clinical and laboratory data to estimate IR in patients with TDM1. The detection of IR in T1DM patients may allow early intervention and possibly impact on future diabetes complications.

## Introduction

Currently, an extensive amount of literature that explores insulin resistance (IR) and adiposity in populations of non-diabetic and type 2 diabetic patients suggests that regional adiposity may be an important determining factor of IR [1–6]. Few investigations have explored these associations in type 1 diabetes mellitus (T1DM). IR is traditionally related to type 2 diabetes (T2DM), but its association with T1DM is also well documented [7–11]. The most probable cause for the lack of studies in this area is the difficulty of directly evaluating IR in this population. In individuals at risk for T1DM, increased IR concomitant to the decrease in beta-cell mass can alter the balance between insulin sensitivity (IS) and secretion which then precipitates hyperglycemia [12]. Thus, this imbalance could result in a more aggressive form of the autoimmune disorder, mediated by immunoinflammatory factors common to both processes, that mediates both IR and the destruction of beta cells, such as TNF-α and IL-6 [13]. These concepts are part of the “Accelerating Hypothesis” [14]. A family history of T2DM and chronic hyperglycemia (glucotoxicity) during the clinical phase of T1DM are associated with decreased peripheral glucose uptake. Other factors may also influence IS, such as age, lean body mass, ethnicity, body fat, weight, physical activity, and drug use [6, 8, 15–17]. In patients with T1DM, the development of coronary artery disease (CAD) occurs decades earlier and with a frequency tenfold higher than in non-diabetic individuals. Although the factors associated with increased risk of CAD in this population are well documented, its pathogenesis remains unclear [18]. IR is related to diabetic nephropathy and CAD [19], and the latter represents one of the major causes of mortality in adult the population with long-lasting T1DM [7, 20, 21]. IR is an independent risk factor for the development of micro and macrovascular diseases in both T2DM and T1DM patients [7, 21].

The hyperinsulinemic-euglycemic clamp technique is the gold standard for the diagnosis of IR [22]. However, the difficulty in performing, high cost of and invasive nature of this procedure limit its large scale use [7, 8]. Some clinicians have proposed indirect and simplified approaches to estimate IR using clinical parameters of the disease and individual characteristics of the patients via mathematical formulas [23–26]. In this context, two formulas that estimate IR have been validated by comparison with the hyperinsulinemic-euglycemic clamp technique and have been used, in spite of some limitations. The first formula associates the estimated glucose disposal rate (eGDR) with the waist/hip (cm) ratio, history of systemic arterial hypertension and the haemoglobin A1c (%) level, which are inversely related to IR [23]. The second formula evaluates the insulin sensitivity score (ISS) based on the waist size (cm), level of glycated haemoglobin (A1c) (%) and triglycerides (mg/dl) [26].

Therefore, the present study aims to assess the presence of IR in patients with DM1 with the eGDR and ISS formulas, estimate the agreement between these formulas, and assess the correlation between clinical and laboratory parameters with IR. This is the first investigation of IR evaluation in a group of Brazilian type 1 diabetic patients using two mathematical formulas and adiposity markers.

## Subjects and methods

This cross-sectional study was performed between January 2011 and June 2012 in Belo Horizonte, Minas Gerais, Brazil. The study included 135 patients with T1DM selected consecutively and followed up at the outpatient care department for diabetic patients at the Endocrinology Service of the Hospital Santa Casa and Hospital das Clínicas of the Universidade Federal de Minas Gerais. T1DM was diagnosed according to the criteria of the American Diabetes Association [27]: onset of the disease before 30 years of age and permanent insulinization beginning less than one year after the diagnosis [21]. Patients with A1c values greater than 11.1% (which were the maximum values used in the two formulas), a pubertal stage less of than 5, the presence of chronic renal failure, infection or pregnancy at the time of data collection were not included in the study. All subjects agreed to participate in the study and signed an informed consent form. The project was approved by the Research Ethics Committees of the institutions involved (Projects CAAE −06121203003-11 and CEP 012/2011).

The anamnesis and physical examinations of the participants were conducted by trained examiners. The following information was obtained from the clinical history: age, gender, duration of T1DM (years), total insulin dose IU/ lean mass (kg), arterial hypertension, physical activity (present in those who performed aerobic activity or resistance for more than 180 minutes per week) and a family history of T2DM. The body weight was measured while patients wore light clothes and no shoes on scales with an accuracy of 100 g. The height was measured with the patient barefoot, standing with feet and heels parallel, and the trunk, shoulders and head touching the stadiometer (accuracy of 0.5 cm). Waist and hip circumferences were measured in centimeters at the lowest curvature between the ribs and the iliac crest and at the area of greatest gluteal protuberance, respectively. The measurement of the systolic and diastolic blood pressure (SBP and DBP, mmHg) was performed in supine and standing positions. Individuals using antihypertensive medications and/or those with a SBP ≥130 mmHg and/or DBP ≥80 mmHg were considered hypertensive. The presence of *acanthosis nigricans* was recorded during the physical examination.

Skin fold (SF) thickness was measured at the triceps, suprailiac and subscapular areas, chest, armpit, abdomen, and thigh. The following parameters were calculated: body mass index (BMI) given by weight (kg)/height^2^ (m), waist/height ratio (cm), waist-hip ratio (W/H), and fat percentage (FP). We used two methods to assess body composition. The first was a dual-energy X-ray absorptiometry (DEXA; DEXA Lunar Expert, model 1081) method. Total body fat mass (kg), free lean bone mass (kg), and percentage of body lean mass were calculated by measuring regional arm lean mass (kg), leg lean mass (kg), and trunk lean mass (kg). The separation between the trunk and legs and arms was made according to the protocol used by Shay *et al.*[23]. One participant was excluded from the analysis due to a lower limb amputation. From the total of 135 patients, 64 (47.4%) were submitted to DEXA. In the second method, we measured the SF thickness three times and used the arithmetic mean of these values. The adipometer (Lange, Cambridge Scientific Industries, USA) used has an accuracy of 0.2 mm. To calculate the SF thickness, we used Jackson and Pollock’s formulas [28, 29]. For men, SF =1.10938 - 0.0008267 (thoracic + abdominal + thigh) +0.0000016 (thoracic + abdominal + thigh)^2^ - 0.0002574; and FP = [(4.95/DC) - 4.50] × 100 [28]. For women SF =1.0994921 - 0.0009929 (triceps + supra-iliac + thigh) +0.0000023 (triceps + supra-iliac + thigh)^2^ - 0.0001392; and FP = (5.01/D4.57) × 100 [29].

Blood collections were performed after fasting for a minimum of 10 hours and abstention from alcoholic beverages for at least 72 hours. Laboratory tests consisted of the following: fasting plasma glycaemia (FPG), total cholesterol, HDL cholesterol (HDL), LDL cholesterol (LDL), triglycerides (enzymatic s3colorimetric method), A1c (determined by immunoturbidimetry and high performance liquid chromatography (HPLC), both standardized by the National Glycohemoglobin Standardization Program (NGSP)), glutamic acid decarboxylase (GAD) antibodies test, and an insulinoma-associated (IA2) antibodies test (performed by radioimmunoassay and C-peptide by the chemiluminescence method).

IR was determined using two formulas that were validated by the hyperinsulinemic-euglycemic clamp technique. The first formula, eGDR, allows estimates of the glucose release rate [23] and is calculated based on clinical and laboratory parameters according to the following equation: eGDR =24.4 - (12.97 × W/H) - (3.39 × AH) - (0.60 × A1c); where W/H is the value of the waist/hip ratio and AH indicates the presence of arterial hypertension (yes = 1, no = 0). The second formula estimates the ISS [26] and is calculated with the equation: LogeIS = 4.64725 - 0.02032 (W, cm) - 0.0977 (A1c, %) - 0.00235 (TG, mg/dl); where Loge IS is the logarithm of IS, W is the waist size, and TG is the triglyceride level.

The results of the eGDR and ISS formulas were separated into quartiles as follows: eGDR 1st quartile (≤6.61), 2nd quartile (6.62 to 8.76), 3rd quartile (8.77 to 10.33), and 4th quartile (>10.33); and ISS 1st quartile (≤6.22), 2nd quartile (6.23 to 8.16), 3rd quartile (8.17 to 9.56), and 4th quartile (>9.56).

Sample size was calculated according to the following parameters: 0.25 prevalence of metabolic syndrome (and hence, IR) in T1DM patients [10, 30], 0.12 estimation error, 5% alpha and 20% power. The estimated sample size was 120 patients for an error tolerance of 0.05. These patients were randomly selected by simple randomization from a pool of 350 DM1 patients that attended the institutions where the research was performed.

Statistical analyses were performed using the computer software IBM SPSS, version 20.0 (SPSS Inc., Chicago, IL, USA) and R version 3.01 (2013-05-16, Vienna, Austria). We used histograms and Shapiro-Wilk tests to verify the normality of continuous data. The Student’s T tests and Mann–Whitney U tests were used to compare means and medians of continuous variables, and the Person’s chi-square test or Fisher’s exact test to compare categorical variables between the men and women. Spearman’s correlation coefficient was used to evaluate the correlation between the IR formulas and markers of adiposity, clinical findings, laboratory parameters and T2DM family history. The original data from the formulas was calculated and divided into quartiles because the formulas do not have cutoff values for comparative analyses. We divided patients into three groups (1st quartile, 2nd + 3rd quartiles and 4th quartile). The Jonckheere-Terpstra test was used to compare medians of continuous variables (without Gaussian normal distribution) between the three groups (1st quartile, 2nd + 3rd quartiles and 4th quartile) and Chi-square test for trends and Fisher’s exact test to compare categorical variables between the three groups (1st quartile, 2nd + 3rd quartiles and 4th quartile). All analyses were performed using p < 0.05 as a statistically significant level. The two formulas in question were compared using the Kappa coefficient. Finally, the association between the 1st quartile of the ISS formula data and the occurrence *acanthosis nigricans* was analyzed using the odds ratio.

## Results

The characteristics of the 135 subjects with T1DM are presented in Table 1, which shows the clinical and laboratory profiles according to gender. The proportion of smokers was higher among men (20.4% vs. 8.2% p = 0.029). A total of 46.6% of men and 33.8% of women were classified as overweight (BMI ≥25 kg/m^2^). In the study population, 71.5% of patients had a positive family history of T2DM. The auto antibodies anti-GAD and anti-IA2 were positive in 87.3% and 25% of women and 92.1% and 16.7% of men, respectively. C-peptide levels were undetectable in 88.1% of women and 88.9% of men.

**Table 1:** **Clinical and laboratory profile of type 1 diabetic patients**

Variables	Male	Female	P value
N =135	58 (43%)	77 (57%)	
Age (years)	30 (17–68)	27 (12–58)	0.139
TDM1 duration (years)	15.5 (1–48)	15.0 (1–49)	0.556
eGDR	8.49 (1.26-11.82)	9.3 (2.35-12.6)	0.055
ISS	7.60 (±2.34)	8.30 (±2.28)	0.090
BMI^†^ (kg/m^2^)	24.0 (19.0-36.0)	24.0 (17.0-46.0)	0.255
Waist (cm)	84.5 (65–119)	78.0 (64–123)	0.001
Hips (cm)	98.0 (80–120)	96.0 (90–150)	0.938
Waist/height ratio	49.3(36.1-70.4)	48.1 (38.5-78.9)	0.81
Waist/hip ratio	0.85 (0.75-1.12)	0.79 (0.67-1.48)	<0.001
Total insulin dose UI/Lean mass (kg)^‡^	0.98 (0.32-3.15)	1.14 (0.19-3.35)	0.085
Total lean mass (kg)^‡^	53.15 (±9.55)	36.82 (41.63)	<0.001
Thoracic-abdominal fat (kg)^‡^	3.63 (1.0-28.65)	6.65 (2.34-17.37)	0.053
Leg fat (kg)^‡^	3.86 (0.85-12.93)	6.81 (2.3-16.34)	<0.001
Arm fat (kg)^‡^	0.738 (0.182-3.743)	1.81 (0.51-6.78)	<0.001
Total lean mass percent^‡^	83.8 (56–95)	68.7 (50–86)	<0.001
Total Fat Percent^‡^	16.24 (5.3-43.6)	33.00 (14.4-50)	<0.001
Thoracic-abdominal total percent^‡^	14.76 (5–68.4)	26.30 (11–50.3)	0.004
Leg fat percent^‡^	18.90 (8.7)	39.27 (8.33)	<0.001
Arm fat percent^‡^	11.1 (4.1-32.6)	38.0 (14.9-59.1)	<0.001
Triceps skinfold (mm)	12.30 (4.33-31.67)	20.67 (8.33-48.67)	<0.001
Scapular skinfold (mm)	15.70 (6.33-41.67)	18.33 (6.33-39.0)	0.035
Total cholesterol (mg/dL)	160 (76–282)	169 (85–332)	0.043
HDL cholesterol (mg/dL)	50 (27–81)	58 (36–124)	0.001
LDL cholesterol (mg/dL)	94.0 (35–178)	98.0 (20–252)	0.419
Triglycerides (mg/dL)	62.5 (26–310)	63 (25–295)	0.697
Systolic arterial pressure mmHg	120 (90–200)	120 (90–160)	0.012
Diastolic arterial pressure mmHg	80 (50–120)	80 (50–100)	0.017
A1c (%)	7.6 (5.4-11)	7.9 (5.8-11.1)	0.328
HbA1c(mmol/mol)	60 (36–94)	63 (40–98)	0.328
Total insulin dose (IU/kg)	55 (12–340)	44 (8–122)	0.036
Physical activity – yes	41 (70.7%)	43 (56.6%)	0.107
Acanthosis – yes*	4 (7.3%)	6 (8.2%)	0.10
Family history of TDM2 – yes	40 (70.2%)	53 (72.6%)	0.845

P value: Person’s chi- square, Fisher’s exact test*, Student’s *T* test, and the Mann–Whitney test for differences between, frequencies, frequencies, means and medians.

^†^Body mass index = weight (kg)/height (m)^2^.

^‡^Patients who underwent densitometry for analysis of body composition (n = 64).

eGDR estimated glucose disposal rate, ISS insulin sensitivity score, BMI body mass index, A1c glycated haemoglobin.

**Figure 1:**
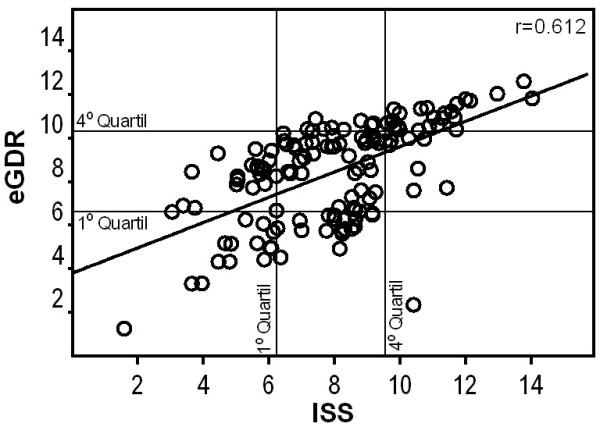
**Scatter plot of the correlation values between the first quartile and fourth quartile of data from the formulas that estimate the glucose disposal rate (eGDR) and the insulin sensitivity score (ISS) and the Spearman correlation coefficient.**

Table 2 shows the correlation analysis between the ISS and eGDR formulas by gender and fat markers, clinical and laboratory parameters, T2DM family history and physical activity. According to the Spearman’s rank correlation coefficient, a moderate positive correlation was found between the values of the two formulas (r = 0.612, p < 0.001) (Figure 1).

**Table 2:** **Spearman linear correlation between eGDR, ISS and clinical and laboratory profiles by gender**

Variables	eGDR		ISS	
	SLC General		SLC General	
	SLC Male	SLC Female	SLC Male	SLC Female
Total lean mass (kg)^§^	−0.121 (0.335)		−0.262 (0.034)	
	0.064 (0.751)	−0.103 (0.533)	−0.443 (0.021)	−0.262 (0.107)
Thoracoabdominal fat (kg)^§^	−0.484 (<0.001)		−0.758 (<0.001)	
	−0.560 (0.002)	−0.478 (0.003)	−0.825 (<0.001)	−0.743 (<0.001)
Leg fat (kg)^§^	−0.068 (0.592)		−0.351 (0.004)	
	−0.296 (0.134)	−0.078 (0.648)	−0.563 (0.002)	−0.374 (0.23)
Arm fat (kg)^§^	−0.381 (0.002)		−0.592 (<0.001)	
	−0.497 (0.08)	−0.390 (0.17)	−0.667 (0.001)	−0.662 (<0.001)
Percentage of total lean mass^§^	0.262 (0.036)		0.436 (<0.001)	
	0.464 (0.015)	0.322 (0.052)	0.659 (0.001)	0.534 (<0.001)
Percent total Fat^§^	−0.268 (0.032)		−0.476 (<0.001)	
	−0.464 (0.015)	−0.349 (0.034)	−0.659 (0.001)	−0.640 (<0.001)
Percentage of thoracoabdominal fat^§^	−0.359 (0.004)		−0.574 (<0.001)	
	−0.369 (0.058)	−0.464 (0.004)	−0.592 (0.001)	−0.731 (<0.001)
Percent fat leg^§^	−0.024 (0.848)		−0.167 (0.188)	
	−0.311 (0.114)	−0.067 (0.695)	−0.487 (0.01)	−0.260 (0.120)
Percent arm fat^§^	−0.224 (0.75)		−0.402 (<0.001)	
	−0.545 (0.003)	−0.326 (0.049)	−0.718 (<0.001)	−0.619 (<0.001)
Triceps skinfold (mm)	−0.62 (0.518)		−0.336 (<0.001)	
	−0.286 (0.44)	−0.906(0.457)	−0.595 (<0.001)	−0.380 (0.002)
Subscapular skinfold (mm)	−0.356 (<0.001)		−0.569 (<0.001)	
	−0.485 (0.001)	−0.374 (0.003)	−0.676 (<0.001)	−0.594(<0.001)
HDL cholesterol (mg/dL)	0.122 (0.158)		0.231 (0.007)	
	0.076 (0.570)	0.079(0.496)	0.197 (0.138)	0.185 (0.108)
Total cholesterol (mg/dL)	−0.035 (0.69)		−0.140 (0.107)	
	0.152 (0.259)	−0.022(0.846)	−0.205 (0.125)	−0.147 (0.201)
BMI^||†^ (kg/m^2^)	−0.373 (<0.001)		−0.721 (<0.001)	
Waist/height ratio^||^	−0.537 (<0.001)		−0.779 (<0.001)	
Age (years)^||^	−0.495 (<0.001)		−0.190 (0.027)	
Diabetes duration (years)^||^	−0.428 (<0.001)		−0.187 (0.03)	
Total dose insulin IU/Lean mass(kg)^§||^	−0.279 (0.0023)		−0.398 (<0.001)	
Acanthosis nigricans - yes^||^	−0.110 (0.218)		−0.258 (0.003)	
LDL cholesterol (mg/dL)^||^	−0.115 (0.187)		−0.154 (0.075)	
Family history of TDM 2^||^ - yes	0.15 (0.868)		−0.096 (0.277)	
Physical activity – yes^||^	0.142 (0.102)		0.024 (0.78)	

P value: Spearman correlation.

^†^Body mass index = weight (kg)/ height (m)^2^.

^§^Patients who underwent bone densitometry for body composition analysis (n = 64).

^||^Variables where there was no significant difference for the gender factor. See Table 1.

The clinical and laboratory characteristics of diabetic patients belonging to the 1st and 4th quartiles according to the eGDR and ISS formulas are presented in detail in Tables 3 and 4. The variables that were significantly different between the 1st quartile and 4th quartile of the eGDR formula data comprise age, duration of diabetes, BMI, thoracic-abdominal fat (absolute and percentage), total dose insulin IU/lean mass (kg), arm fat, waist/height ratio and scapular skinfold. The variables that were significantly different between the 1st quartile and 4th quartile of the ISS formula data comprise age, duration of diabetes, BMI, total insulin dose per lean mass, total fat percentage, arm fat, leg fat, thoracoabdominal fat (absolute and percentage), scapular skinfold, triceps skinfold, total lean mass (absolute and percentage), percentage of total fat, and hip and waist/height ratio. A moderate positive association was found between the first and 4th ISS quartiles regarding HDL values and the presence of *acanthosis nigricans*.

**Table 3:** **Clinical and laboratory characteristics of type 1 diabetic patients compared by eGDR quartiles**

Variables	eGDR 1 ^st^ quartile	eGDR 2 ^nd^ and 3 ^rd^ quartiles	eGDR 4 ^th^ quartile	P value
Patients	n = 33 (24.4%)	n = 69 (51.1%)	n = 33 (24.4%)	
Gender (Male)	18 (54.5%)	29 (42.0%)	11 (33.3%)	0.083
Age (years)	37 (20–68)	29 (12–58)	23 (12–55)	< 0.001
Duration of diabetes (years)	21 (2–48)	14 (1–49)	10 (0–27)	< 0.001
BMI^†^ (kg/m^2^)	25 (19–35)	24 (17–46)	21 (18–28)	0.001
Waist/height ratio	52.6 (40.5-70.4)	50 (38.7-78.8)	44.2 (36.1-52.2)	< 0.001
Total insulin dose IU/Lean mass (kg)^§^	1.13 (0.46-3.15)	1.14 (0.52-3.35)	0.91 (0.19-1.63)	0.059
Total lean mass (kg)^§^	40 (32.9-64.2)	41 (30.1-81.1)	38.5 (30.1-58.0)	0.656
Thoracoabdominal fat (kg)^§^	9.38 (2.3-28.7)	6.34 (1.0-20.1)	4.71 (1.7-12.5)	0.002
Leg fat (kg)^§^	5.40 (0.9-11.0)	6.29 (0.9-12.9)	5.89 (1.8-16.3)	0.901
Arm fat (kg)^§^	1.98 (0.3-6.8)	1.46 (0.2-3.7)	1.03 (0.3-4.8)	0.017
Total lean mass percentage^§^	0.70 (0.5-0.9)	0.72 (0.6-1.0)	0.78 (0.5-0.9)	0.108
Total fat percentage^§^	0.29 (0.07-0.5)	0.28 (0.05-0.40)	0.21 (0.08-0.49)	0.103
Thoracoabdominal fat percentage^§^	0.29 (0.09-0.54)	0.25 (0.05-0.41)	0.19 (0.08-0.68)	0.02
Leg fat percentage^§^	0.31 (0.06--0.50)	0.31 (0.06-0.48)	0.30 (0.11-0.60)	0.852
Arm fat percentage^§^	0.32 (0.55-0.59)	0.30 (0.4-0.43)	0.26 (0.45-0.56)	0.122
Triceps skinfold (mm)	15.5 (6.6-30.0)	17.8 (4.3-48.7)	19.0 (5.3-28.7)	0.730
Scapular skinfold (mm)	19.5 (7.7-39.0)	17.3 (6.3-41.7)	13.8 (6.3-26.0)	0.001
Acanthosis (yes)*	2 (6.5%)	7 (10.8%)	1 (3.1%)	0.432
Physical activity (yes)	17 (53.1%)	43 (62.3%)	24 (72.7%)	0.103
Family history of DM2 (yes)	24 (72.7%)	43 (66.2%)	26 (81.3%)	0.457
Total insulin dose (IU/day)	48 (20–340)	52.5 (17–161)	36 (8–85)	0.041
Total Cholesterol (mg/dl)	166 (104–322)	166 (85–240)	165 (76–250)	0.853
HDL (mg/dl)	56 (27–87)	52 (32–124)	58 (32–107)	0.474
LDL (mg/dl)	100 (57–252)	96 (20–178)	93 (35–150)	0.357

P-value: Jonckheere-Terpstra test, Chi-square for trends, Fisher Exact Test* for comparisons of medians, frequencies and frequencies.

^†^Body mass index = weight (kg)/height (m)^2^.

^§^Patients who underwent densitometry for the analysis of body composition (n = 64).

**Table 4:** **Clinical and laboratory characteristics of type 1 diabetic patients compared by ISS quartiles**

Variables	ISS 1 ^st^ quartile	ISS 2 ^nd^ and 3 ^rd^ quartiles	ISS4 ^th^ quartile	P value
Patients	n = 34 (25.2%)	n = 68 (50.4%)	n = 33 (24.4%)	
Gender (Male)	18 (52.9%)	30 (44.1%)	10 (30.3%)	0.063
Age (years)	32 (15–68)	27 (12–62)	25 (15–55)	0.016
Duration of diabetes (years)	17 (1–49)	16 (2–43)	10 (0–27)	0.020
BMI (kg/m^2^)^†^	27.5 (19–46)	24 (17–30)	21 (18–26)	< 0.001
Waist/height ratio (cm)	56.8 (40.5-78.8)	49.0 (38.7-61.9)	43.7 (36.1-51.7)	< 0.001
Total insulin dose IU/Lean Mass (kg)^§^	1.43 (0.53-3.35)	1.05 (0.46-1.77)	0.92 (0.19-1.63)	0.002
Total lean mass (kg)^§^	0.63 (0.5-0.9)	0.74 (0.5-1.0)	0.79 (0.7-0.9)	0.001
Thoracoabdominal fat (kg)^§^	13.13 (7.2-28.7)	6.28 (1.0-13.6)	3.4 (1.5-6.9)	< 0.001
Leg fat (kg)^§^	7.10 (3.6-12.9)	5.45 (0.9-16.3)	4.76 (1.8-10.4)	0.011
Arm fat (kg)^§^	2.65 (1.2-6.8)	1.36 (0.2-4.9)	0.816 (0.2-2.2)	0.001
Total lean mass percentage^§^	0.63 (0.5-0.9)	0.74 (0.5-1.0)	0.79 (0.7-0.9)	0.001
Total fat percentage^§^	0.37 (0.18-0.50)	0.25 (0.05-0.49)	0.20 (0.08-0.34)	< 0.001
Thoracoabdominal fat percentage^§^	0.33 (0.22-0.50)	0.21 (0.05-0.41)	0.16 (0.06-0.68)	< 0.001
Leg fat percentage^§^	0.35 (0.14-0.48)	0.33 (0.06-0.60)	0.29 (0.11-0.49)	0.237
Arm fat percentage^§^	0.39 (0.16-0.59)	0.28 (0.04-0.56)	0.18 (0.04-0.41)	0.001
Triceps skinfold (mm)	21.7 (9.7-26.6)	15.7 (4.3-30.0)	15.0 (5.3-25.7)	0.003
Scapular skinfold (mm)	24.3 (14.7-41.7)	16.0 (6.3-36.0)	12.0 (6.3-23.7)	< 0.001
Acanthosis (yes)*	6 (19.4%)	3 (4.6%)	1 (3.1%)	0.013
Physical activity (yes)	19 (57.6%)	43 (63.2%)	22 (66.7%)	0.447
Family history of DM2 (yes)	25 (78.1%)	44 (66.7%)	24 (75%)	0.783
Total insulin dose (IU/day)	60 (34–340)	46 (17–111)	36 (8–85)	< 0.001
Total cholesterol (mg/dL)	169 (117–282)	167 (76–332)	162 (76–250)	0.268
HDL (mg/dL)	46 (27–87)	57 (35–107)	56 (32–124)	0.004
LDL (mg/dL)	99 (49–178)	96 (20–252)	88 (35–150)	0.127

P-value: Jonckheere-Terpstra test, Chi-square for trends, Fisher Exact Test* for comparisons of medians, frequencies and frequencies.

^†^Body mass index = weight (kg)/height (m)^2^.

^§^Patients who underwent densitometry for the analysis of body composition (n = 64).

The variables that were strongly concurrent between the 1st and 4th quartiles for both formulas comprise BMI, waist/height ratio, subscapular skinfold, total dose insulin IU/lean mass(kg), age, diabetes duration and thoracoabdominal fat (absolute and percentage).

In the studied population, 14 (10.4%) patients presented results calculated by both formulas that corresponded with the 1st quartile, suggesting IR, and 63 (85.7%) patients were overweight or obese. The agreement between the 1st quartiles of the eGDR and ISS formulas was slight-to-moderate (Kappa = 0.226), and the agreement among the four quartiles of the two formulas was strong (Kappa = 0.679). The individuals with IR classified in the 1st quartile by the ISS formula had a higher chance of presenting with *acanthosis nigricans* (OR = 5.58, 95% CI = 1.46-21.3).

## Discussion

According to the classic definition of T1DM, individuals with a total deficiency of insulin are characterized as T1DM patients. During the last two decades there has been an increase in the overweight population of type 1 diabetic patients, and the prevalence of overweight patients has tripled from the 1980s to the 1990s [31], following the trend of the general population [16]. In our study population, 46.6% of men and 33.8% of women were classified as overweight (BMI ≥25 kg/m^2^). Among the patients who underwent DEXA, we found an increased percentage of total body fat in both men and women, specifically in the groups classified into the 1st quartile by the formulas. In this group, the percentage of total and thoracoabdominal fat, subscapular skinfold (SF), and the waist/height ratio, which are markers of visceral fat, were increased significantly compared to the other quartiles of both formulas. Forty-four percent of young Hispanic Americans with T1DM from the SEARCH study were overweight or obese [32], suggesting a new phenotype in a group of patients that were considered lean until that time. These results also confirmed previous observations in T1DM patients showing that fat stored in the abdomen is associated with an increased risk of cardiovascular disease. Our data are in agreement with the results of a study by Shay *et al.*[24] that showed associations between total and regional adiposity, determined by DEXA. The negative association between insulin sensitivity in T1DM and regional and overall adiposity measures improved the prediction of the eGDR and ISS equations for identifying individuals with T1DM at risk for IR. This further supports the relationship established between general obesity and IR in the studied population. The phenotypical change in the individuals with T1DM is most likely related to the augmentation of the use of insulin, irregular food habits, long time periods spent in sedentary activities, insufficient physical activities and, primarily, genetic backgrounds.

The eGDR and ISS formulas were associated with the following general clinical features: BMI, thoracoabdominal fat, subscapular SF and waist/height ratio. The results of many studies show that IR is related to waist circumference, which, despite limitations, is a useful clinical marker of abdominal adiposity. The association of the formulas with visceral fat deposits was strong in our study. Uncertainty still exists regarding the best way to measure adiposity and evaluate metabolic risks in T1DM patients. DEXA is quickly becoming the preferred technique to evaluate body fat distribution, but the high accuracy provided by this method is associated with increased operational costs. Although DEXA is clinically useful to quantify regional adiposity, current studies suggest that this method is not necessary for the estimation of IR in the general population of individuals with T1DM [24]. More useful clinical markers of obesity, such as BMI, and abdominal adiposity, such as waist and waist/height ratio, have exhibited strong associations with IR. Our results support the regular use of these simple and inexpensive measures to evaluate the risk of IR in T1DM patients.

Our results provide evidence for an association between the formulas used to determine IR and insulin dose per lean body mass in type 1 diabetic patients. Available evidence suggests that the insulin dose by lean mass and by body surface may be associated with IR [33]. Similar to the increased values of obesity and triglyceride markers, the presence of arterial hypertension, low HDL levels and *acanthosis nigricans* are related to central adiposity and IR [34]. In the group of patients analyzed using the IR formulas, in addition to the positive associations regarding the previously mentioned variables, a strong negative correlation between the amount of thoracoabdominal fat and the ISS value (r = −0.758 p < .001) was observed, which provides further evidence that an increased amount of fat stored in the abdomen could be a risk factor for the development of IR. Furthermore, ISS was also significantly associated with *acanthosis nigricans* (a clinical marker of IR), triceps SF, percentage of total fat, percentage of lean mass, and HDL suggesting its enhanced usefulness in the classification of IR patients. This most likely occurs because the ISS formula includes the triglyceride level, which is an important factor associated with of IR. We found associations between age and duration of diabetes in years with IR in both formulas, but the association was stronger with the eGDR formula. A possible explanation for these associations is that hypertension, a component of the formula, is more prevalent in T1DM patients after several years of the disease, although other factors were independently associated with increased risk of hypertension in T1DM as older age, male sex, family history of hypertension, greater baseline body mass index, weight gain, and higher albumin excretion rate [35]. Reaven first drew attention to the association of insulin resistance, obesity, high plasma triglycerides and low HDL cholesterol with hypertension in the type 2 diabetes. We found the same associations for the patients classified into the first quartiles by both formulas for type 1 diabetics [36].

The following limitations of our study should be taken into account. The current design was cross-sectional and did not allow the establishment of a temporal relationship between IR, regional adiposity, and the development of diabetes complications. The percentage of patients who underwent DEXA was approximately 50% of those included in the study. This occurred due to logistical difficulties. We found no correlation between physical activity, history of T2DM and serum levels of LDL, unlike some authors who showed a correlation with family history of T2DM and serum levels of LDL [17]. However, we found an inverse correlation between IR and lean mass, an important marker of physical activity. Physical activity plays an important role in delaying disease progression and is inversely related to mortality in T1DM patients [37]. The use of a simplified questionnaire concerning physical activity may have been a source of bias. In poorly controlled diabetic patients, IR can also result from glucotoxicity because high blood glucose levels increases IR [10]. It is well known that good glycaemia control reduces weight gain and possibly decreases cardiovascular risk factors [38, 39]. Our population is characterized by miscegenation. However, when our findings were compared to those of European populations, which are ethnically different, they did not differ. Findings similar to our results were found in a Polish study in which IR was evaluated by euglycaemic clamp in 202 patients with T1DM. The authors found associations between the same variables: BMI, subscapular SF, total insulin dose, total body fat, blood pressure, A1c, waist size, triglycerides and age [24]. We found no association between other regional adiposity markers and the values of the formulas; however, the evaluations in the Polish study were based only on SF thickness, and no densitometry measures were performed.

Longitudinal studies are needed to elucidate the causal relationship between increasing adiposity and the development of IR, CAD and nephropathy in populations with T1DM. The determination of the role of IR in the natural course of the disease and therapies that reduce IR, such as changes in lifestyle, weight control, the use of metformin [40] and control of oxidative stress, could avoid vascular damage in these patients [41].

In summary, our data, similar those of other studies, suggest that we can determine the presence of IR among type 1 diabetic patients by using clinical and laboratory elements because performing hyperinsulinemic-euglycemic clamp is impractical in population studies. In our analysis, data regarding total and regional body fat improved the estimation of IS. As stated by other authors, the analysis of IR risk factors is important for the overall trend of searching for a simple method that could be used in population studies and clinical practice [42].
